# COGENT (COlorectal cancer GENeTics): an international consortium to study the role of polymorphic variation on the risk of colorectal cancer

**DOI:** 10.1038/sj.bjc.6605338

**Published:** 2009-11-17

**Authors:** I P M Tomlinson, M Dunlop, H Campbell, B Zanke, S Gallinger, T Hudson, T Koessler, P D Pharoah, I Niittymäkix, S Tuupanenx, L A Aaltonen, K Hemminki, A Lindblom, A Försti, O Sieber, L Lipton, T van Wezel, H Morreau, J T Wijnen, P Devilee, K Matsuda, Y Nakamura, S Castellví-Bel, C Ruiz-Ponte, A Castells, A Carracedo, J W C Ho, P Sham, R M W Hofstra, P Vodicka, H Brenner, J Hampe, C Schafmayer, J Tepel, S Schreiber, H Völzke, M M Lerch, C A Schmidt, S Buch, V Moreno, C M Villanueva, P Peterlongo, P Radice, M M Echeverry, A Velez, L Carvajal-Carmona, R Scott, S Penegar, P Broderick, A Tenesa, R S Houlston

**Affiliations:** 1 Molecular and Population Genetics, Nuffield Department of Medicine, University of Oxford, Wellcome Trust Centre for Human Genetics, Roosevelt Drive, Oxford OX3 7BN, UK; 2 Institute of Genetics and Molecular Medicine, University of Edinburgh, MRC-HGU, Western General Hospital, Crewe Road South, Edinburgh EH4 2XU, UK; 3 Public Health Sciences, University of Edinburgh, Edinburgh EH89AG, UK; 4 The Ontario Institute for Cancer Research, The MaRS Center, 101 College St, Suite 800, Toronto, Ontario, Canada M5G 1L7; 5 The University of Ottawa Faculty of Medicine, 101 Smythe Rd, Ottawa, Ontario, Canada K1H 8L6; 6 Cancer Care Ontario, 620 University Ave., Toronto, Ontario, Canada M5G 2L7; 7 Samuel Lunenfeld Research Institute, Mount Sinai Hospital and University of Toronto, 600 University Ave., Toronto, Ontario, Canada M5G 1X5; 8 Department of Oncology, University of Cambridge, Cambridge, UK; 9 Department of Medical Genetics, Genome-Scale Biology Research Program, Biomedicum 9, University of Helsinki, Helsinki, Finland; 10 German Cancer Research Center, Heidelberg, Germany; 11 Department of Molecular Medicine and Surgery, Karolinska Institutet, CMM02, Stockholm S17176, Sweden; 12 LCCI Biomarker Laboratory, Ludwig Institute for Cancer Research, PO Box 2008, Royal Melbourne Hospital, VIC 3050, Australia; 13 Department of Pathology, Leiden University Medical Center, ZA LEIDEN 2333, The Netherlands; 14 Departments of Human and Clinical Genetics, Leiden University Medical Center, ZA LEIDEN 2333, The Netherlands; 15 Laboratory of Molecular Medicine, Human Genome Center, Institute of Medical Science, The University of Tokyo, Tokyo, Japan; 16 Department of Gastroenterology, Institut de Malalties Digestives i Metabòliques, Hospital Clínic, Centro de Investigación Biomédica en Red de Enfermedades Hepáticas y Digestivas (CIBERehd), IDIBAPS, University of Barcelona, Barcelona, Catalonia, Spain; 17 Fundacion Publica Galega de Medicina Xenomica (FPGMX), CIBERER, Genomic Medicine Group-University of Santiago de Compostela, Santiago de Compostela, Galicia, Spain; 18 The University of Hong Kong, Pokfulam Road, Hong Kong, China; 19 Department of Genetics, University Medical Center Groningen, University of Groningen, P.O. Box 30.0001, Groningen 9700 RB, the Netherlands; 20 Institute of Experimental Medicine, Academy of Sciences of the Czech Republic, Videnska 1083, 14200 Prague 4, Czech Republic; 21 Division of Clinical Epidemiology and Aging Research, German Cancer Research Center, Im Neuenheimer Feld 280, Heidelberg 69120, Germany; 22 Department of General Internal Medicine, University Hospital, Schleswig-Holstein, Campus Kiel, Schittenhelmstraße 12, Kiel 24105, Germany; 23 POPGEN Biobank, University Hospital Schleswig-Holstein, Campus Kiel, Schittenhelmstrasse 12, Kiel 24105, Germany; 24 Department of General and Thoracic Surgery, University Hospital Schleswig-Holstein, Campus Kiel, Arnold-Heller-Strasse 3, Kiel 24105, Germany; 25 Institut für Community Medicine, University Hospital Greifswald, Walther-Rathenau-Strasse 48, Greifswald 17487, Germany; 26 Klinik für Innere Medizin A University Hospital Greifswald, Friedrich-Loeffler-Strasse 23a, Greifswald 17487, Germany; 27 Klinik für Innere Medizin C, University Hospital Greifswald, Ferdinand-Sauerbruch-Strasse, Greifswald 17487, Germany; 28 IDIBELL-Catalan Institute of Oncology and University of Barcelona, Av Gran Via 199, L’Hospitalet, Barcelona 08907, Spain; 29 Centre for Research in Environmental Epidemiology (CREAL), Municipal Institute of Medical Research (IMIM-Hospital del Mar) and CIBER Epidemiología y Salud Pública (CIBERESP), Doctor Aiguader, Barcelona 88 E-08003, Spain; 30 Fondazione IRCCS Istituto Nazionale Tumori, and Fondazione IFOM, Istituto FIRC di Oncologia Molecolare, Milan, Italy; 31 Departamento de Biología, Universidad del Tolima, Barrio Altos de Santa Helena, Ibague, Tolima, Colombia; 32 Departamento de Patología, Hospital Pablo Tobon Uribe, Calle 78 B No. 69-240, Medellín, Colombia; 33 Faculty of Health, School of Biomedical Sciences, University of Newcastle, NSW, Australia; 34 Section of Cancer Genetics, Institute of Cancer Research, 15 Cotswold Rd, Sutton, Surrey SM2 5NG, UK

**Keywords:** colorectal cancer, association, polymorphism

## Abstract

It is now recognised that a part of the inherited risk of colorectal cancer (CRC) can be explained by the co-inheritance of low-penetrance genetic variants. The accumulated experience to date in identifying these variants has served to highlight difficulties in conducting statistically and methodologically rigorous studies and follow-up analyses. The COGENT (COlorectal cancer GENeTics) consortium includes 20 research groups in Europe, Australia, the Americas, China and Japan. The overarching goal of COGENT is to identify and characterise low-penetrance susceptibility variants for CRC through association-based analyses. In this study, we review the rationale for identifying low-penetrance variants for CRC and our proposed strategy for establishing COGENT.

## Background

Although inherited susceptibility underlies ∼35% of variance in colorectal cancer (CRC) risk (Lichtenstein *et al*, 2000), high-penetrance germline mutations account for <6% of cases (Aaltonen *et al*, 2007). Much of the remaining variation in genetic risk is likely to be a consequence of the co-inheritance of multiple low-penetrance variants, some of which are common.

The ‘common-disease common-variant’ model of CRC implies that association analyses based on scans of polymorphic variants should be a powerful strategy for identifying low-penetrance susceptibility alleles. This assertion has recently been vindicated by genome-wide association (GWA) studies, which have provided robust evidence for several common low-risk variants influencing CRC risk (Tomlinson *et al*, 2005, 2007; Broderick *et al*, 2007; Zanke *et al*, 2007; Houlston *et al*, 2008; Jaeger *et al*, 2008; Tenesa *et al*, 2008). Although the risk of CRC associated with each of these common variants is individually modest, they make a significant contribution to the overall disease burden by virtue of their high frequencies in the population. Moreover, by acting in concert with each other, they have the potential to significantly affect an individual's risk of developing CRC. Hence, this class of susceptibility alleles is potentially of public health importance, allowing risk stratification within populations. One benefit for risk prediction between population subgroups is that it could enable tailoring of the invasiveness or frequency of large bowel screening, eventually leading to a reduction in mortality and even incidence through secondary prevention. Finally, the identification of new risk variants may identify new cancer pathways that made lead, in time, to the development of new prevention or treatment strategies for CRC.

To facilitate the study of predisposition to CRC, we established COGENT (COlorectal cancer GENeTics), an international consortium with the goal of identifying and characterising low-penetrance genetic variants that predispose to CRC. In this study, we review the rationale for studying low-penetrance susceptibility to CRC and our proposed strategy for COGENT.

### Difficulties in conducting methodologically rigorous association studies

To date, most association studies based on the candidate gene approach have only evaluated a restricted number of polymorphisms, primarily in genes implicated in the metabolism of dietary carcinogens and protection of DNA from carcinogen-induced damage. Reports from these studies have largely been disappointing, with numerous positive associations initially from analyses of small case–control series being unconfirmed by subsequent analyses. Only a minority of studies have reported case–control data on the same variants, allowing pooling of data (Table 1). Although *P*-values from meta-analyses of such studies provide limited support for the role of variants in *MTHFR* (Huang *et al*, 2007; Hubner and Houlston, 2007), *CCND1* (Tan *et al*, 2008), *GSTT1* (de Jong *et al*, 2002), *XPC* (Zhang *et al*, 2008), *NQO1* (Chao *et al*, 2006) and *NAT2* (Chen *et al*, 2005), such analyses should be interpreted with caution even if publication bias is ignored. Use of false-positive report probability value (FPRP) (Wacholder *et al*, 2004), which integrates the earlier probability for association and statistical power, provides one method for assessing the robustness of summary estimates derived from pooled analyses. Although earlier probabilities are partly subjective, influenced by previous findings and experimental evidence with regard to the known impact of variants, the earlier probability for variants in candidate genes is unlikely to be better than 1 in 1000 (or 0.001) (Thomas and Clayton, 2004). Imposing a ‘best case’ value less than 0.001 and stipulating an odds ratio of 1.2 for associations, it is noteworthy that the likelihood of any of the variants being associated with CRC risk is not high (i.e., FPRP >0.2 suggested to be appropriate for summary analyses (Wacholder *et al*, 2004)). Hence, despite much research, until the advent of GWA studies, few, if any, definitive susceptibility alleles for CRC have been unequivocally identified through association studies. The accumulated experience to date has served to highlight the difficulties in conducting statistically and methodologically rigorous association studies to identify new cancer predisposition loci. The main issues are summarised below:

1. The increase in CRC risk conferred by any common polymorphic variant is almost certainly small (i.e., typical relative risk ∼1.2). The inherent statistical uncertainty of case–control studies involving just a few hundred cases and controls severely constrains study power to reliably identify genetic determinants conferring modest, but potentially important, risks.
2. As of the large number of polymorphisms in the genome, false-positive associations are inevitably more frequent than true-positive associations when testing large numbers of generic markers (especially when using off-the-shelf SNP arrays), even if studies are conducted in a scientifically rigorous manner. Hence, associations need to attain a high level of statistical significance to be established beyond reasonable doubt. For this reason, in GWA studies, a *P*-value threshold of 5.0 × 10^−7^ has been advocated and is generally considered to be appropriate for genome-wide significance.
3. Positive associations need to be replicated in independent case–control series to further limit the type 1 error rate. However, to increase power, the allelic architecture of the population from which these case–control series are ascertained needs to have similar ancestry and, ideally, the same linkage disequilibrium (LD) structure.
4. It should be recognised that cancers such as CRC are somewhat heterogeneous with respect to aetiology and biology. Specifically for CRC, colonic and rectal disease may have different risk factors and have a varied spectrum of somatic mutations and epimutations. It must thus be recognised that a given variant may not affect the risk of all histological forms of CRC. The power of any analysis stratified by histology is therefore limited because of the smaller numbers of cases in each group.
5. Careful attention must be paid to population stratification as a source of confounding, because cancer rates and allele frequencies vary with race/ethnicity. This is one possible explanation for some of the false-positive associations reported in literature.
6. Epidemiological risk factor data should ideally be taken into consideration to allow the examination of interactions between known aetiological factors (e.g., dietary risk factors) and genetic risk variants. As very large sample sizes are probably needed to detect interactions, the power of these types of analyses in the association studies reported to date has been extremely limited.
7. Rare germline polymorphisms may be more highly penetrant and have significance for individuals, although the population-attributable risk may be low. Extreme examples include the previously identified mutations in DNA repair enzymes and Lynch Syndrome. Only through genotyping and sequencing of large numbers of individuals can additional rare variants that confer important individual risk be identified. Advances in sequencing technology make this feasible.

### Characteristics of low-penetrance variants

Most studies aimed at identifying low-penetrance alleles for cancer susceptibility have been based on a candidate gene approach formulated on preconceptions of pathology pertaining to the role of specific genes in the development of CRC. However, without a clear understanding of the biology of predisposition, the choice of suitable genes for the disease is inherently problematic, and very few susceptibility loci for CRC have been identified that adopt this strategy. An unbiased approach to genetic analysis is therefore required.

The availability of high-resolution LD maps and hence of comprehensive sets of tagging SNPs that capture most of the common sequence variation allows GWA studies for disease associations to be efficiently conducted. This approach is agnostic in that it does not depend on previous knowledge of function or presumptive involvement of any gene in disease causation. Moreover, it minimises the probability of failing to identify important common variants in hitherto unstudied loci (i.e., genes and regulatory regions).

**Figure 1:**
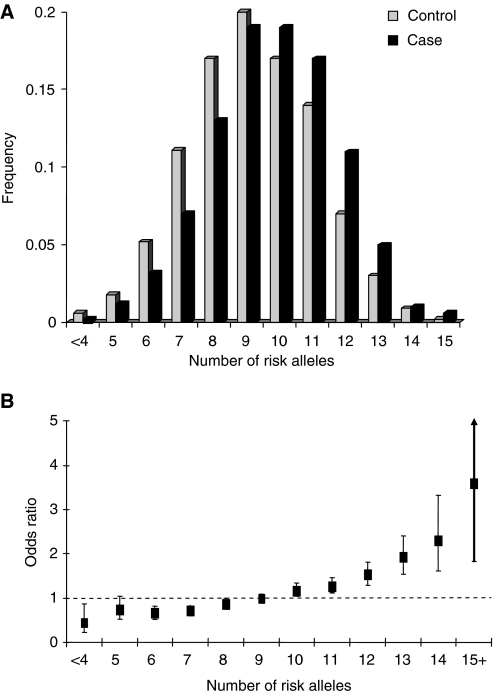
Polygenic model of colorectal cancer susceptibility. (**A**) Distribution of risk alleles for CRC, cases (black bars) and controls (grey bars); (**B**) Plot of the increasing ORs for CRC with increasing number of risk alleles. The ORs are relative to the median number of risk alleles; Vertical bars correspond to 95% confidence intervals. Data from Houlston *et al* (2008).

Three GWA studies of CRC have so far been reported and 10 independent loci shown conclusively to be associated with CRC risk: 8q24.21, 11q23, 18q21.1 (*SMAD7*), 8q23.1 (*EIF3H*), 15q (*GREM1*), 19q13.1 *(RHPN2)*, 20q12.3, 14q22.2 (*BMP4*), 16q22.1 (*CDH1*) and 10p14 (Tomlinson *et al*, 2005, 2007; Broderick *et al*, 2007; Zanke *et al*, 2007; Houlston *et al*, 2008; Jaeger *et al*, 2008; Tenesa *et al*, 2008). Risks associated with each of the common variants at each of these loci are modest (ORs 1.1–1.3; Table 2) and there is little evidence of interactive effects. With homozygous risk variants conferring twice the heterozygote risk, the distribution of risk alleles follows a normal distribution in both case and controls, with a shift towards a higher number of risk alleles in affected individuals consistent with a polygenic model of disease predisposition (Figure 1A). Figure 1B shows the ORs relative to the median number of risk alleles. Individuals with 15+ risk alleles have at least a three-fold increase in risk compared with those with a median number of risk alleles.

Data from these GWA studies and results from similar gene discovery efforts in other tumours are proving to be highly informative with regard to the allelic architecture of cancer susceptibility in general. The number of common variants that account for more than 1% of inherited risk is very low and only a small proportion of the heritability of any cancer can be explained by currently identified loci. Estimates of the contribution of currently identified loci to excess familial risk of CRC may be conservative, as there may be imperfect tagging surrogates for true aetiological loci. Multiple causal variants may also exist at each locus, including low-frequency variants with significantly larger cumulative effects on risk. Few of the observed disease-associated variants are coding variants, with many of the loci mapping to regions bereft of genes or protein-encoding transcripts. It is likely that much of the common variation in cancer risk is mediated through sequence changes influencing gene expression, perhaps in a subtle manner, or through effects on pathway components mitigated by functional redundancy.

## Future directions

### Prospects for identifying additional common variants

The power of existing GWA studies to identify common alleles conferring risks of 1.2 or greater (such as the 8q24 variant) is high. Hence, there are unlikely to be many additional CRC SNPs with similar effects for alleles with frequencies >0.2 in populations of European ancestry. Recent studies have had low power to detect alleles with smaller effects and/or MAFs <0.1. By implication, variants with such profiles are likely to collectively confer substantial risk because of their multiplicity or sub-maximal LD with tagging SNPs. The tagging SNPs used for GWA studies capture on an average ∼80% of common SNPs in the European population (i.e., *r*^2^>0.8), but only ∼12% of SNPs with MAFs of 5–10% are tagged at this level, limiting the power to detect this class of susceptibility allele. GWA-based strategies are not configured optimally to identify low-frequency variants with potentially stronger effects or to identify recessively acting alleles. Nor are current arrays formatted ideally to capture copy number variants or other structural variants such as small-scale insertions or deletions, which may affect CRC risk. It is therefore highly likely that a large number of low-penetrance variants remain to be discovered. This assertion is supported by the continued excess of associations observed over those expected in studies reported to date. Further efforts to expand the scale of GWA meta-analyses, in terms of both sample size and SNP coverage, and to increase the number of SNPs taken forward to large-scale replication may identify additional variants for CRC.

Analyses of most GWA studies have so far been primarily directed towards identifying single locus SNP associations. It is possible that analyses based on haplotypes of markers may identify ‘rarer’ disease alleles that may be present on rare haplotypes missed by single SNP analyses. Under certain circumstances, especially in which interaction effects are large and main effects are small, gene–gene interactions may be detected where no locus with a main effect has been identified (Marchini *et al*, 2005). Multi-locus approaches may therefore be the focus of future experiments as they may yield greater power to detect associations under certain genetic models.

### Identifying causal variants

Validated tagSNPs are highly unlikely to directly cause CRC. Identifying the causal variant from a tagSNP that is statistically associated with disease is difficult. Although blocks of LD allow the efficient survey of the genome, they hamper fine mapping of the disease-associated region. Different ethnic groups are likely to have different LD block patterns and they can therefore be used to refine the location of a disease susceptibility locus before resequencing and functional analyses. The usefulness of this approach depends on the size of the study and SNP allele frequencies in different ethnic groups. In some of these populations, lower environmental risk exposure with lower CRC, incomplete case ascertainment and recording tools, as well as absence of large sample sets, are other challenges.

### Incorporating non-genetic risk factors into risk models

Colorectal cancer risk will probably be determined by complex interactions between the various genetic and lifestyle/dietary risk factors. Epidemiological studies have established several dietary risk factors for colorectal neoplasia; these include low vegetable and high meat consumption (especially processed meat), and micronutrient deficiency and excessive alcohol intake. There is a weaker association between CRC, smoking and lack of physical activity. Common genetic variants are likely to interact with these environmental–lifestyle risk factors to modify risk. Furthermore, common gene variants will have a role in determining the effectiveness of chemoprevention agents such as non-steroidal anti-inflammatory drugs, hormone replacement therapy and micronutrient supplementation.

In assessing the interplay between inherited and non-genetic risk factors, analyses using different population cohorts will be highly informative. Wider comparisons between the population genetics of different ethnic groups have shown that SNP allele frequencies can vary greatly among ethnic groups, principally as a result of founder effects and genetic drift. Indeed, some SNPs may be informative in one population and not in another. At least in principle and probably in practice, some variants may have stronger or weaker effects on disease, depending on environment or general genetic background, as observed in inbred lines of mice.

Identification of the interaction between genetic variants and environmental risk factors is contingent on very large data sets ideally from different population cohorts, something only achievable through multi-centre collaborations. Even with such collaborative efforts, incorporating environmental risk factor data into models of predisposition is likely to be a serious challenge as, although ethnicity can be defined through genotype, environmental background is harder to standardise.

### Inherited prognostic and predictive variants

In addition to influencing the risk of developing CRC, inherited genetic host factors are likely to influence the natural course of the disease. As a potential prognostic factor, the concept of germline variation imparting inter-individual variability in tumour development, progression and metastasis is receiving increasing attention. Compared with breast cancer, studies of the impact of germline variation on CRC prognosis have been more limited. Prognostic studies have generally examined the same candidate genes that are considered to have a role in predisposition. Genetic variation affecting inter-individual disease expression may influence the later stages of malignancy rather than early events associated with an inherited predisposition. Variants in growth factor, apoptosis or immune surveillance signalling pathways, for instance, might not cause CRC initiation but could have a substantial effect on the outcome of established disease. Chemotherapy response and toxicity may be related to germline genotype. Linking GWA data to patient outcome provides an attractive strategy for identifying new prognostic markers. It is essential to impose appropriate statistical thresholds and conduct replication analyses to avoid reporting false positives.

## Rationale for the cogent consortium

The recognition that low-penetrance alleles contribute to the inherited risk of CRC represents a major advance in our understanding. In view of the above-noted issues, over the last few years, collaborations have been steadily developing between groups in the United Kingdom, Canada, the Americas, Holland, Germany, Finland, Spain and Australia that are engaged in ongoing searches for low-penetrance CRC variants through association-based studies. What initially began from relatively loose affiliations centred around work on specific projects has begun to crystallise into a more formal collaborative network after replication analyses of two published GWA studies. To continue and expand collaboration, a meeting was held at the University of Leiden, the Netherlands, in January 2009 to review ongoing association studies. There assembled an international team of researchers with expertise encompassing genetic epidemiology, statistical genetics, gene mapping, biology, molecular genetics, pathology and diagnosis and clinical management of CRC.

There was a consensus among participants that many of the challenges inherent in this field can best be addressed by international cooperative efforts, and the group unanimously decided to establish a CRC association consortium. An invitation to join COGENT that was subsequently extended to other groups known to be performing CRC association studies was well received. At present, 20 groups that are performing case–control genetic association studies have joined COGENT (Table 3). The eligibility criterion for inclusion is the involvement in a case–control study based on at least 500 cases and 500 controls sampled from the same population. The sample size limit aims to ameliorate the potential statistical, biological and technological/methodological confounding effects of small sample sizes (Moonesinghe *et al*, 2008). Collectively, over 48 000 cases and 43 000 controls have so far been accrued by COGENT researchers (Table 3).

In each of the study centres, collection of samples and of clinico-pathological information has been undertaken with informed consent and relevant ethical review board approval in accordance with the tenets of the Declaration of Helsinki. Material transfer agreements have already been used between partners to allow for sharing of individualised data, and similar procedures will be adopted for future collaborative work.

Data pooling provides a very cost-effective approach to achieve an adequate power for subgroup analyses, which are unlikely to have sufficient sample sizes in a single study. Several potential problems need to be considered at the stage of data pooling. Given that individual studies have different data formats, covariates from individual studies will be agreed upon and compiled into a common set of variables relevant to specific projects. Study data sets sent from different centres will be checked for outliers, aberrant distribution, inadmissible values and inconsistencies before pooling to ensure data accuracy. Systematic variation between centres in terms of genotyping will be assessed globally using principal components and on a per-SNP basis. Discrepancies can be cross-verified with study centres.

COGENT represents the first international collaborative study seeking to comprehensively understand the impact of low-penetrance susceptibility to CRC and to describe the genetic landscape of the disease. The immediate goal of the group is to work together collaboratively to study polymorphisms that were previously associated with CRC risk and to plan for future high-quality studies. Past productive collaboration has laid the groundwork for these future studies centred initially on the expansion of discovery and replication of GWA studies, with biological analyses of variants and epidemiological studies as longer-term aims.

## Figures and Tables

**Table 1:** Polymorphisms reported to be statistically significant in meta-analyses

**Polymorphism**	**Risk group**	**MAF/at risk frequency**	**OR (95% CI)**	***P*-value**	**No studies/cases**	**Power, OR=1.2**	**FPRP @ prior of 0.001**
*CCND1*-G870A	GA *vs* GG	0.12–0.64	1.18 (1.06–1.32)	0.0031	12/4614	61%	0.86
*GSTT1*-null	Null *vs* present	0.21–0.44	1.37 (1.17–1.60)	8.1 × 10^−5^	11/1490	5%	0.60
*MTHFR*-V222A	TT *vs* CC	0.32–0.40	0.83 (0.75–0.93)	0.0007	25/12 261	47%	0.74
*MTHFR* A1298C	CC *vs* CA+AA	0.29–0.22	0.81 (0.69–0.96)	0.012	14/4764	37%	0.98
NAT-acetylator	Rapid *vs* slow	0.32–0.77	1.08 (1.00–1.16)	0.04	18/6741	99%	0.97
*NQO1*-Pro187Ser	CT+TT *vs* CC	0.11–0.23	1.18 (1.02–1.35)	0.02	5/1637	60%	0.96
*XPC* lys939Gl	CA *vs* AA	0.65–0.61	1.32 (1.11–1.56)	0.001	2/1060	13%	0.90

Abbreviations: CI=confidence interval; FPRP=false-positive report probability; MAF=minor allele frequency; OR=odds ratio.

Adapted from Dong *et al* (2008) and Hubner and Houlston.

**Table 2:** The 10 loci associated with colorectal cancer risk identified from GWA studies (Tenesa and Dunlop, 2009)

**Gene/locus**	**Chromosome**	**SNP**	**Effect size (odds ratio)**	**Allele frequency**	**Population attributable risk (%)**
—	8q24	rs6983267	1.21 (1.15–1.27)	0.51	9.7
*GREM1*	15q13	rs4779584	1.26 (1.19–1.34	0.18	4.5
*SMAD7*	18q21	rs4939827	1.18 (1.12–1.23	0.52	8.6
—	11q23	rs3802842	1.12 (1.07–1.17)	0.29	3.4
*EIF3H*	8q23	rs16892766	1.25 (1.19–1.32)	0.07	1.7
—	10p14	rs10795668	1.12 (1.10–1.16)	0.67	7.4
*BMP4*	14q21	rs4444235	1.11 (1.08–1.15)	0.46	4.8
*CDH1*	16q22	rs9929218	1.10 (1.06–1.12)	0.71	6.6
*RHPN2*	19q13	rs10411210	1.15 (1.10–1.20)	0.90	11.9
*BMP2*	20q12	rs961253	1.12 (1.08–1.16)	0.35	4.0

Abbreviation: GWA=genome-wide association.

**Table 3:** Number of CRC cases and controls currently established by COGENT consortium members

			**Number of subjects**	
**European**	**Study name**	**General setting**	**Cases**	**Controls**
Institute of Cancer Research, UK	NSCCG (National Study of Colorectal Cancer) (Penegar *et al*, 2007)	Population-based UK study. Spouse controls from NSCCG (Penegar *et al*, 2007) and GELCAPS (Genetic Lung Cancer Predisposition Study) (Eisen *et al*, 2008)	12 976	6000
Edinburgh University, UK	COGS (Colorectal Cancer Genetics Susceptibly Study) (Porteous *et al*, 2003)	Population-based incident case series aged <55 at diagnosis. Population-based controls	1012	1012
	SOCCS (Scottish Colorectal Cancer Study) (Theodoratou *et al*, 2008)	Population-based incident case series; Scotland, UK	3000	3000
Oxford University, UK	CORGI (Colorectal Tumour Gene Identification Consortium) (Kemp *et al*, 2006)	Cases with family history of CRC ascertained through clinical genetics centres in the UK. Spouse controls with no personal or family history of CRC	940	965
	VICTOR – post-treatment stage of a Phase III, randomised double-blind, placebo-controlled study of rofecoxib (VIOXX) in colorectal cancer patients after potentially curative therapy (Kerr *et al*, 2007)	Samples from a closed clinical trial	910	—
	QUAZAR2 – multicentre international study of capecitbine+/−bevacizumab as adjuvant treatment of CRC	UK blood donors	139	376
Cambridge University, UK	SEARCH (Studies of Epidemiology and Risk Factors in Cancer Hereditary)	Population-based case–control study; Cambridge, UK	3000	3000
Barcelona and Santiago, Spain	EPICOLON Consortium (Castells and Andreu, 2007)	Population-based case–control study; Spain	2000	2000
Barcelona, Spain	ENTERICOS (Disinfection by-products and other Environmental, genetic and molecular determinants of colorectal cancer – subproductos de la desinfección y otros determinantes ambientales, genéticos y moleculares del cáncer colorectal en España)	Case–control study of CRC to evaluate the increased risk associated with chronic DBP exposure through ingestion, inhalation and dermal absorption	500	500
	Bellvitge Case–Control Study		370	325
University of Helsinki, Finland	FCCPS (Finnish Colorectal Cancer Predisposition Study) (Salovaara *et al*, 2000)	Population-based study; South-eastern Finland	1440	2000
Karolinska Institute, Sweden		Unselected cases ascertained through 12 hospitals serving the Stockholm-Gotland and Uppsala-Örebro health-care regions in Sweden. Blood donor controls	3000	3000
German Cancer Research Centre (DKFZ): on behalf of German HNPCC consortium	German HNPCC consortium (Frank *et al*, 2008)	Familial non-HNPCC cases recruited through German HNPCC consortium, principally through six hospitals of Bochum, Bonn, Dresden, Düsseldorf, Heidelberg and Munich/Regensburg. Controls: unrelated and ethnicity- and age-matched blood donors recruited by the Institute of Transfusion Medicine and Immunology, Faculty of Mannheim, Germany	1000	1000
University of Keil and Greifswald, Germany	POPGEN (Population Genetic Cohort) from Schleswig-Holstein, north Germany (Krawczak *et al*, 2006; Schafmayer *et al*, 2007). SHIP (Survey of Health in Pommerania) from east and north-east Germany (Volzke *et al*, 2005)	Population-based biobank projects	2720	2720
German Cancer Research Centre	ESTHER (Epidemiologische Studie zu Chancen der Verhütung, Früherkennung und optimierten Therapie chronischer Erkrankungen in der älteren Bevölkerung)	Population-based biobank project	670	670
Institute of Experimental Medicine, Academy of Science, Czech Republic	—	Unselected CRC cases mainly recruited from nine oncological departments (Prague, Benesov, Brno, Liberec, Ples, Pribram, Usti nad Labem and Zlin) (Tulupova *et al*, 2008). Controls hospital patient and blood donors	1300	3000
University of Groningen, the Netherlands	SCOPE study (de Jong *et al*, 2005)	Unselected CRC cases, hospital patient controls from the Netherlands	774	1000
University of Leiden, the Netherlands		Unselected CRC cases. Controls ascertained through genetic testing programmes for non-cancer-related conditions	1500	1500
Fondazione IRCCS Istituto Nazionale Tumori, Milan, Italy		Unselected CRC cases, population controls	700	1200
				
*Australia*				
Ludwig Institute for Cancer Research, Melbourne	Victorian Cancer Biobank	Population-based biobank project	1000	500
The University of Newcastle, New South Wales	Hunter Family Cancer Service	Population-based collection of cases and controls from the Hunter Region of New South Wales	600	3000
				
*The Americas*				
Ibague, Colombia. Universidad del Tolima		Unselected CRC cases, population-based controls	500	700
Toronto, Canada	OFCCR (Ontario Familial Colorectal Cancer Registry) (Cotterchio *et al*, 2000)	Population-based case–control study; Ontario	1257	1336
				
*Asia*				
University Hong Kong Medical Centre, China	UHKMC series	Unselected CRC cases, hospital patient controls	2000	2000
University of Tokyo, Japan	Biobank Japan	Population-based biobank project	6000	6000
				
Total			49 308	46 804

Abbreviations: COGENT=COlorectal cancer GENeTics; CRC=colorectal cancer.
