# COGENT (COlorectal cancer GENeTics): an international consortium to study the role of polymorphic variation on the risk of colorectal cancer

**DOI:** 10.1038/sj.bjc.6605518

**Published:** 2010-01-19

**Authors:** I P M Tomlinson, M Dunlop, H Campbell, B Zanke, S Gallinger, T Hudson, T Koessler, P D Pharoah, I Niittymäki, S Tuupanen, L A Aaltonen, K Hemminki, A Lindblom, A Försti, O Sieber, L Lipton, T van Wezel, H Morreau, J T Wijnen, P Devilee, K Matsuda, Y Nakamura, S Castellví-Bel, C Ruiz-Ponte, A Castells, A Carracedo, J W C Ho, P Sham, R M W Hofstra, P Vodicka, H Brenner, J Hampe, C Schafmayer, J Tepel, S Schreiber, H Völzke, M M Lerch, C A Schmidt, S Buch, V Moreno, C M Villanueva, P Peterlongo, P Radice, M M Echeverry, A Velez, L Carvajal-Carmona, R Scott, S Penegar, P Broderick, A Tenesa, R S Houlston

**Correction to**: *British Journal of Cancer* **102**, 447–454. doi: 10.1038/sj.bjc.6605338

Upon online publication of the above article on 17 November last year (and now this issue), it was noted that two of the author's names, I Niittymäki and S Tuupanen, had been incorrectly spelled owing to incorrect submission to the journal. They are now included in the full author list, above, with their correct spelling.

The authors would like to apologise for this mistake.

